# A new consensus for evaluating CDKL5/STK9‐dependent signalling mechanisms

**DOI:** 10.15252/embj.2018100848

**Published:** 2018-10-30

**Authors:** Patrick A Eyers

**Affiliations:** ^1^ Department of Biochemistry Institute of Integrative Biology University of Liverpool Liverpool UK

**Keywords:** Cell Adhesion, Polarity & Cytoskeleton, Genetics, Gene Therapy & Genetic Disease, Post-translational Modifications, Proteolysis & Proteomics

## Abstract

Mutation or inactivation of CDKL5 kinase is associated with a human neurodevelopmental condition commonly referred to as CDKL5 deficiency disorder.^§^ Two recent phosphoproteomics studies identify the first physiological substrates of mammalian CDKL5 and evaluate functional consequences of their phosphorylation and its loss in cells lacking functional CDKL5, highlighting potential roles for this kinase in regulating neuronal microtubule dynamics.

Protein phosphorylation plays a fundamental role in basic cell biology, and the protein kinase superfamily has become a goldmine for potential drug‐intervention strategies due to kinase mutation or dysregulation in human diseases. However, it is often (conveniently) forgotten that mechanistic information is lacking for much of the human kinome, and this includes an absence of basic knowledge pertaining to regulatory mechanisms and substrate specificity (Wilson *et al*, 2018). A case‐in‐point are the cyclin‐dependent kinase‐like (CDKL) kinases, a small family of five poorly characterised and structurally distinct Ser/Thr protein kinases from the CGMC evolutionary branch of the kinome, which contains GSK3α, MAPK and CDK families (Canning *et al*, 2018). Mammalian CDKL5 (also known as STK9) is widely expressed in cells, where it is targeted to a variety of subcellular structures (Barbiero *et al*, 2017; Oi *et al*, 2017). Of particular interest, inactivation of the X‐linked CDKL5 gene, or disease‐associated mutations commonly found in the N‐terminal CDKL5 catalytic domain, causes a neurodevelopmental disorder termed CDKL5 deficiency disorder (CDD; Tao *et al*, 2004; Weaving *et al*, 2004), which has some overlapping features with Rett syndrome (Scala *et al*, 2005) and West syndrome (Kalscheuer *et al*, 2003).^§^

Writing in *The EMBO Journal*, two groups simultaneously reveal the first physiological substrates for CDKL5, including potential new biomarkers for reporting cellular CDKL5 activity. Using distinct, but highly complementary mass spectrometry (MS)‐based phosphoproteomics approaches, the laboratories of John Rouse and Matthias Trost (Muñoz *et al*, 2018), and Sila Ultanir (Baltussen *et al*, 2018) evaluate functional consequences of CDKL5‐catalysed substrate phosphorylation and its loss in cells lacking functional CDKL5. These findings will be critical for understanding microtubule dynamics and cilium‐based signalling in CDD cell models, including neurons from patients with pathological CDKL5 mutations.

Both teams exploited MS to establish, in as unambiguous a way as is possible, the direct substrates of CDKL5 in cellular models. Although the approaches taken were different, they (happily) converge on a subset of substrates related both through shared subcellular function(s) and conserved consensus site(s) of CDKL5 phosphorylation. Muñoz and colleagues initially employed a clean CRISPR knockout/in approach in human U2OS cells ± CDKL5, which was coupled to a quantitative (6‐plex TMT) phosphoproteomic workflow (Muñoz *et al*, 2018). This high‐quality, internally controlled experimental strategy revealed nearly 200 sites of intracellular protein phosphorylation (including CDKL5 autophosphorylation) whose increase was statistically relevant when CDKL5 was added back to CDKL5 genome‐edited cells. In a distinct, no‐less impressive approach, Baltussen and colleagues developed CDKL5 chemical proteomics, employing purified catalytically active CDKL5‐F89A/C152A, an analogue‐sensitive (as) version selectively using the bulky ATP analogue benzyl‐ATPγS, to thiophosphorylate potential substrates in a mouse brain lysate (Baltussen *et al*, 2018). After affinity‐based purification of phosphorylated substrate‐derived peptides, mass spectrometry identified multiple potential CDKL5 substrates, including murine microtubule‐associated targets MAP1S (two sites of phosphorylation), EB2 and ARHGEF2 (Baltussen *et al*, 2018) and, independently, human MAP1S, CEP131 and DLG5 (Muñoz *et al*, 2018). At a single stroke, these consolidating approaches reveal, for the very first time, new signalling functions of CDKL5 that are relevant to microtubule assembly, cilia‐based signalling and perhaps polarity‐based cellular networks (Fig 1A).

**Figure 1:**
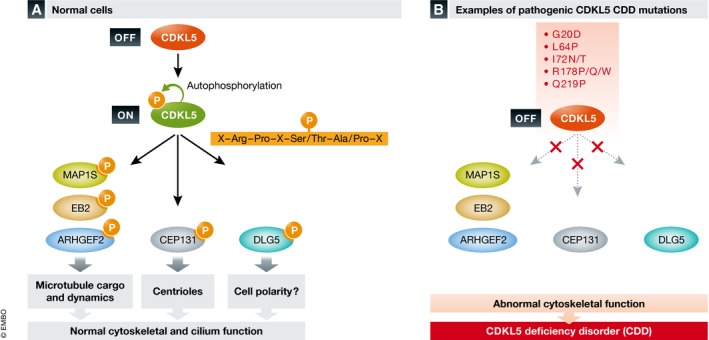
Physiological CDKL5 substrates control cytoskeletal function (A) Through an unknown signalling mechanism, CDKL5 catalytic activity is switched on via Tyr (auto)phosphorylation in the activation segment Thr‐Glu‐Tyr motif. Active CDKL5 phosphorylates various cellular pools of substrates in the consensus Arg‐Pro‐X‐Ser‐Ala, leading to phosphorylation‐dependent changes in microtubule dynamics and cargo transportation. (B) In cells containing pathology‐associated CDKL5 mutations, CDKL5 exhibits much reduced activity and fails to phosphorylate various target proteins. Changes in cytoskeletal dynamics, including loss of targeted EB2 and MAP1S phosphorylation, promote abnormal microtubule functions and neuronal phenotypes associated with CDD.

In the case of the microtubule‐binding protein MAP1S, a biological function for phosphorylation was confirmed in elegant murine follow‐up experiments, exploiting phospho‐specific MAP1S antibodies to demonstrate that phosphorylation on Ser812 (equivalent to human Ser900) was impaired in microtubule binding *in vitro*, consistent with a significant reduction in microtubule dynamics in living cortical neuron dendrites derived from CDKL5 knockout mice (Baltussen *et al*, 2018). In order to probe signalling with additional reagents, both studies made use of additional phospho‐specific antibodies. For example, the phosphorylation of MAP1S at Ser812 and EB2 at Ser222 is both markedly reduced in CDKL5 knockout mice regardless of age, validating phosphoproteomics data and establishing CDKL5 activity markers in cells based around proteins that modulate microtubule trafficking and dynamics (Fig 1B). Perhaps the icing on the cake, however, is the demonstration that endogenous EB2 phosphorylation (as judged by a phospho‐specific EB2 antibody able to recognise an identical phospho‐epitope in human and mouse EB2) is severely reduced (by ~80%) in neurons derived from CDD patient fibroblasts after reprogramming into iSPCs (Baltussen *et al*, 2018). Importantly, many of the antibodies validated in the two studies can now be employed, or re‐engineered as higher‐affinity antibodies, to serve as diagnostic tools for quantifying cellular activity of CDKL5, most obviously to evaluate CDKL5 biology and diagnose CDKL5 phenotypes, but also for therapeutic approaches being developed to normalise CDKL5 expression (see below).

Another exciting outcome from these studies is the finding that catalytically active CDKL5 phosphorylates Ser/Thr in a sharply defined, specific amino acid consensus sequence in its target substrates (Fig 1A), as has been documented for many other protein kinases. A harmonious minimal CDKL5 phosphorylation consensus emerging from both papers is the amino acid sequence Arg‐Pro‐X‐Ser/Thr‐Ala/Pro, in which the Ser or Thr residue (heavily biased 85:15% Ser:Thr) is phosphorylated, adjacent to a C‐terminal residue (commonly Ala or Pro). These latter findings, confirmed biochemically with model peptide substrates, agree with phosphoproteomics data sets, which demonstrated CDKL5 autophosphorylation sites in Ser/Pro motifs alongside a dominant general Arg‐X‐X‐Ser motif (Muñoz *et al*, 2018). Although the CDKL5 consensus overlaps to some extent with known sites of phosphorylation for other “basophilic” kinases (e.g. Arg‐X‐X‐Ser‐Ala for several AGC kinases) and MAPK/CDKs (minimally Ser/Thr‐Pro), the absolute conservation of a Pro at −2, and a specific requirement for Arg at −3, may well be diagnostic for CDKL5 (and perhaps other CDKL orthologues). Further studies employing active CDKL1/2/3/4 proteins (which, somewhat surprisingly, do not appear to phosphorylate any of the physiological CDKL5 substrates identified in this study) should open up these unstudied human kinases to careful scrutiny using similar chemical genetic or genome editing approaches.

The functional consequences of pathological CDKL5 mutations were previously uncertain, with evidence for inhibition and preservation of activity, alongside Tyr phosphorylation in the TEY activation segment (Bertani *et al*, 2006). By quantifying the reduction of CDKL5‐dependent MAP1S and CEP131 phosphorylation in cells expressing specific CDD mutants, and by using a new MAP1S S900 peptide assay to assess immunoprecipitated CDKL5 activity, Muñoz and colleagues now unequivocally identify them as loss‐of‐function mutations that inactivate CDKL5.

Intriguingly, biochemical analysis demonstrates that CDKL5 also possesses an inherent ability to phosphorylate itself on a conserved regulatory residue (Tyr171) in the activation segment Thr‐Glu‐Tyr motif. This autophosphorylation event appears to be intimately associated with an active kinase conformation when evaluated *in vitro*, and might also be relevant for generating active CDKL5 in cells (Bertani *et al*, 2006). Interestingly, a Thr‐X‐Tyr motif is conserved in all five human CDKL protein kinases; its mutation to an Asp‐X‐Glu phosphomimetic version in CDKL1‐5 prior to crystallographic studies (Canning *et al*, 2018) provides supporting evidence of the importance of this motif for driving structural dynamics. However, how (or even whether) CDKL5 phosphorylation is regulated at this motif in cells remains to be established, since the mechanism through which it is triggered is currently unknown. It is intriguing to speculate that CDKL5 is not only able to control its own activity, but that mutations in CDD patients might also lead to a loss in CDKL5 catalytic output through a lack of activation‐loop Tyr phosphorylation. The best‐known “dual‐specificity” protein kinases are the MAPKK/MEK family of MAPK activators, which catalyse dual phosphorylation of a Thr‐X‐Tyr motif (Thr‐Glu‐Tyr for MEK1/2) in the activation segment of cognate MAPK substrates. A concerted autoactivation mechanism in CDKL5 suggests that it possesses (biochemical) properties of a dual‐specificity protein kinase, and opens up this mechanistic event in CDKL kinases for further analysis. In the related MAPKs, dual phosphorylation of the Thr‐X‐Tyr motif massively increases catalytic activity, whereas Tyr15 phosphorylation is strongly associated with inhibition at a motif distinct from the activation segment in CDKs. It will therefore be interesting to evaluate whether CDKL5 autophosphorylation on Tyr (and/or Thr) also occurs on the other four CDKL proteins (where the human sequence is Thr‐Asp‐Tyr, rather than Thr‐Glu‐Tyr), or whether Tyr is also phosphorylated on cellular proteins distinct from CDKL5. Finally, CDKL5 Tyr autophosphorylation does not rule out the presence of distinct Tyr or dual‐specificity kinases lying “upstream” of CDKL5; putative activators include the interacting dual‐specificity tyrosine phosphorylation‐regulated kinase DYRK1A (Oi *et al*, 2017), which was previously shown to contribute to CDKL5 subcellular targeting.

A central impact from this work is the discovery of the very first physiological CDKL5 substrates and the emergence of a framework for the development of multiple approaches to study and measure (and eventually normalise) CDKL5 activity in systems relevant to CDD, such as the brain. Further detective work will be required to uncover the complete set of cellular CDKL5 substrates (many more being predicted in these phosphoproteomics studies) and to decipher regulatory networks associated with CDKL5 catalytic output that are central to physiological function and underlie CDKL5 loss‐of‐function in CDD. Unravelling the molecular mechanisms of CDKL5 activation and inactivation could also help link subset(s) of the CDKL5 network that contribute to disease phenotypes to potential therapeutics. In terms of targetable outputs, factors that control CDKL5 activation include pharmacological agents linked to activity of the NMDA receptor (Tramarin *et al*, 2018), whose inhibition by experimental neuronal depolarisation has a significant experimental effect on EB2 phosphorylation at pS222 (Baltussen *et al*, 2018). Several avenues might also be explored in related areas of cell biology, most notably any links between human CDKL5 and the ciliopathy‐associated protein CENP131, which is associated with centriolar stability and the DNA damage response. Centriolar and cilium‐based analysis also provide fascinating new potential models for functional CDKL5 analysis. For example, human CDKL5 has recently been demonstrated to localise to the cilium, and effects of patient‐derived mutations have been modelled in *C. elegans*, where CeCDKL1 (most similar to human CDKL1/4) regulates cilium length, and *Chlamydomonas*, where CrCDKL5 is also required for controlling aspects of cilial dynamics (Canning *et al*, 2018).

Finally, very recent studies have begun to analyse small molecule kinase inhibitors, including the pre‐clinical GSK3α inhibitor tideglusib, which exhibits some promise for restoring memory function in immature CDKL5 knockout mice, although the specific intracellular target that mediates these effects is unknown (Fuchs *et al*, 2018). Further work is now required to link the specific subset(s) of phosphorylated CDKL5 targets that contribute to disease phenotypes, and to understand the function of the long C‐terminal region, which is much longer in CDKL5 than other CDKL proteins, and presumably plays a key role in its physiological function. Rational drug design using recently available CDKL5 structures and small molecule screening data (Canning *et al*, 2018) could provide new ways to activate mutated (inactive) CDKL5 mutants, alongside the development of genetic therapies, or even protein‐replacement therapies (Trazzi *et al*, 2018).
